# Understanding the Interaction Determinants of CAPN1 Inhibition by CAST4 from Bovines Using Molecular Modeling Techniques

**DOI:** 10.3390/molecules190914316

**Published:** 2014-09-11

**Authors:** Han-Ha Chai, Dajeong Lim, Eunkyoung Jung, Bong-Hwan Choi, Yong-Min Cho

**Affiliations:** 1 Animal Genome & Bioinformatics Division, National Institute of Animal Science, RDA, Suwon 441-706, Korea; 2 Insilicotech Co. Ltd., C-602 Korea Bio Park, 694-1 Sampyeong-Dong, Bundang-Gu, Seongnam-Shi 463-400, Gyeonggi-Do, Korea

**Keywords:** CAPN1 (μ-calpain), CAST4 (the fourth repetitive calpain-inhibition domain of calpastatin), Bovine CAPN1/CAST4 system, computer-aided molecular design, anti-BVDV drug

## Abstract

HCV-induced CAPN activation and its effects on virus-infected cells in a host-immune system have been studied recently. It has been shown that the HCV-nonstructural 5A protein acts as both an inducer and a substrate for host CAPN protease; it participates in suppressing the TNF-α-induced apoptosis response and downstream IFN-induced antiviral processes. However, little is known regarding the disturbance of antiviral responses generated by bovine CAPN activation by BVDV, which is a surrogate model of HCV and is one of the most destructive diseases leading to great economic losses in cattle herds worldwide. This is also thought to be associated with the effects of either small CAPN inhibitors or the natural inhibitor CAST. They mainly bind to the binding site of CAPN substrate proteins and competitively inhibit the binding of the enzyme substrates to possibly defend against the two viruses (HCV and BVDV) for anti-viral immunity. To devise a new stratagem to discover lead candidates for an anti-BVDV drug, we first attempted to understand the bovine CAPN-CAST interaction sites and the interaction constraints of local binding architectures, were well reflected in the geometry between the pharmacophore features and its shape constraints identified using our modeled bovine CAPN1/CAST4 complex structures. We propose a computer-aided molecular design of an anti-BVDV drug as a mimetic CAST inhibitor to develop a rule-based screening function for adjusting the puzzle of relationship between bovine CAPN1 and the BVDV nonstructural proteins from all of the data obtained in the study.

## 1. Introduction

Calpains (CAPNs) are types of proteinases, which represent almost 2% of all gene products [1], and they belong to Ca^2+^-dependent cysteine-endoproteinases, which hydrolyze the internal α-peptide bonds between the amino acids of substrate proteins in the presence of Ca^2+^ ions for enzyme activation. The physiological functions of CAPNs themselves and their endogenous inhibitor calpastatin (CAST) have been major targets in medicine and biotechnology due to their multi-faceted virtual roles. On the other hand, functional disturbances in the coding-level constraints of the CAPN isoforms, their activity states and pathological conditions, are associated with various diseases, and imbalance in their functions is thought to cause many diseases, including Alzheimer’s disease, stroke, and brain trauma with plaque formation by CAPN1, and in the eye, cataract formation caused by CAPN2, and a lens-specific variant of CAPN3 under their over-activation [2,3,4,5,6,7,8,9,10]. Many disease-associated cellular processes lead to protease destabilization and aggregation (amyloid diseases, limb-girdle muscular dystrophy caused by defects in CAPN3). Protease aggregation may also lead to a decrease in its activity and may elicit an inordinate immunological response against inflammation sites [11,12,13]. This suggests that the physiological importance of CAPN is not addressed enough, when viewed in light of the cause-and-effect relationships of calpainopathies. Effectors or executors of Ca^2+^-overload have been targeted for the development of therapeutic reagents to regulate or stabilize of the CAPN system.

Bovine CAPN1 has been a focus of our work. We have taken a strong interest in bovine CAPN1 due to the strong anti-Bovine Viral Diarrhea Virus (BVDV) capability of CAPN small inhibitors. BVDV is a major bovine pathogen. It is a member of the well-characterized *Pestivirus* as a surrogate model virus for the hepatitis C virus in the same *Flaviviridae* family of viruses. BVDV infection is one of the most destructive diseases, leading to major economic losses in cattle herds worldwide. In the United States alone, there is an average loss of $10 to $40 per calving [14]. Nevertheless, there are no the direct BVDV infection remedies. Recent studies [15,16,17,18] have suggested that the nonstructural 5A (NS5A, a serine phosphoprotein) protein of the hepatitis C virus (HCV) acts as both an inducer and a substrate for the host CAPN by activating the cysteine protease by the perturbation of intracellular Ca^2+^ ions, which stimulate its protease activity producing the NS5A protein in the HCV-infected cell. The common ability of nonstructural proteins of the BVDV and HCV to avoid the antiviral response generated by the host innate immune system results from their multi-functional biological role in mediating essential steps of the viral life cycle and modulating virus-induced immunosuppression. In other words, a dramatic activation of the natural CAPN has been known to be responsible for the activation of NF-κB (nuclear factor-κ light chain enhancer of activation B cells) transcription factor with the NF-κB-dependent expression of pro-inflammatory cytokines and adhesion molecules in addition to calcineurin/NFAT pathways in T-cell activation [9,10,19]. In contrast, CAPN activation by the HCV-NS5A protein, which suppresses apoptosis of the infected cell, inhibits TNF-α (tumor necrosis factor-α) induced apoptosis; however, pharmacological inhibition of CAPN reactivates the apoptosis response of the NS5A expresses [20]. If looks upside down, the cleavage of the NS5A protein by the host CAPN may be likely to a modulator as a posttranslational modification on the NS5A processing, are supported by the HCV infection experiments [15,16,17,18,19,20], where completely blocked tyrosine phosphorylation of IκBα (nuclear factor of κ-light polypeptide gene enhancer in B-cells inhibitor, α) induced by the HCV-NS5A protein and the subgenomic replicons in subsequent NF-κB activation, were regulated by the small CAPN inhibitors (ALLM, ALLN, and MDL-28170). Furthermore, the NS5A protein directly constrains the activity of the host antiviral protein PKR (an interferon-induced double-stranded RNA-activated protein kinase that brings about the NF-κB activation process) via association with an IFN sensitivity-determining region (ISDR) located at the center of the NS5A molecules, resulting in IFN-resistances and downstream IFN-induced antiviral processes. The effects of small CAPN inhibitors against the BVDV-NS5A protein can be indirectly inferred from the characteristics of BVDV and HCV; they share similar viral life cycles and host immune responses [21,22] to their infections; therefore, BVDV has been considered a good model virus for HCV. However, some similarities and differences in the prototypic representation between the BVDV and HCV have not been considered. The facts of both the surrogate virus model for HCV infection and the relationship between the NS5A and NS5B (the nonstructural 5B protein has RNA-dependent RNA polymerase activity) of BVDV proteins that correlate with the NS5A activates transcription factors of NF-κB and STAT-3 (Signal transducer and activator of transcription-3) as well as host CAPN. Thus the STAT-3 binds to the NS5B protein and thus, targets the IFN-α JAK/STAT signaling for anti-viral immunity. This led us to investigate the link between the BVDV-NS5B protein and the host CAPN protease. Recently, we characterized the impact of basic aromatic analogues on anti-BVDV activity that have been targeted for the NS5B polymerase by the complementary 2D-QSAR and MFA models [23]. In a previous study, we suggested that the polarity and the polarizability of a series of arylazoenamine derivatives were very important for optimal inhibitory activity of the BVDV- NS5B polymerase and would be further optimized for prevention of HCV, and other single-strand RNA viruses in the *Falviviridae* family. The bovine CAPN/CAST complex system should give priority to the potential host factor target as a stratagem against BVDV infection to enhance the bovine response to control BVDV owing to its high pathway redundancy for the host in wide expression in infected tissues. More importantly, these facts motivated us to apply the CAPN inhibitors in the virtual screening of new lead candidates for the design of an anti-BVDV drug to improve the efficiency of immune-mediated inhibition of BVDV-infected cells. As a result, there has been substantial interest in the action site of the BVDV-induced CAPN enzyme in modulation of the virus-infected cell apoptosis in the evasion of innate immune response [24] by the BVDV pathogen.

To develop a new stratagem for the bovine BVDV pathogen/host CAPN environment, we first attempted to understand the target molecular characteristics, such as the activation and inactivation of CAPN as well as, the regulation of CAPN activity by its natural inhibitor CAST with a focus on CAPN1 member specificities. If CAPN-CAST interaction sites can be determined, antibodies specific to those sites and small inhibitors for the specific substrate proteolysis [4,5] (in particular, the BVDV-NS5A protein) can be screened *in silico* to irreversibly block increase in the viral immunological evasion mediated by the host CAPN protease. The CAPN activity in the virus-infected cell should be tightly controlled by the inhibitor CAST, which has a high affinity for the activated form of CAPN. The CAST inhibition modes are far more specific than the substrate-competitive processes present in most cell types. The binding sites and modes of the human CAPN-CAST action mechanism are used as a model from the design to the development of therapeutic protein drugs. There are more than 50 currently known endogenous and exogenous inhibitors of CAPN. These can be divided into two groups: peptidomimetics at the active site (the protease core cleft) that mimic fragments of its natural CAST inhibitor (peptidyl epoxides, aldehyde, and α-ketoamides) and non-peptide inhibitors at other allosteric sites of domain DIV or DVI [6,25,26,27,28,29,30,31,32,33,34]. Unfortunately, the active site inhibitors (target the CAPN protease core) are generally nonspecific for CAPN family members or other cysteine proteases. Indeed, peptide-based inhibitors exhibit poor pharmacokinetics in contrast to allosteric inhibitors, and have limitations for inclusion in the classical CAPN subgroup (the CAPNs: 1, 2, 3, 8, 9, 11, 12, 13 and 14). The limitations are due to differences in the structural architectures of the whole enzyme between members of the classical and non-classical CAPN subgroups. Therefore, there is a high potential to repurpose these small CAPN inhibitors like the CAST inhibitor’s imitations for the development of BVDV antiviral treatments. In addition, interaction constraint predictions of the binding sites of the CAST inhibitor to the CAPNs proteases will warrant further investigation into their potential for anti-BVDV applications such as mimic CAST analog and therapeutic targets of anti-BVDV to the host CAPNs proteases.

Our previous study [35] involving the structure prediction of bovine CAPN1/CAST complex contributed towards understanding how the fourth CAST inhibitory domain (CAST4) interacts with CAPN1 to inhibit its protease activity using the predicted complex model by focusing on the interaction patterns with the bovine CAPN1 trace residues, which were concentrated in both conserved and class-specific residues across the CAPN1 and CAPN2 subgroups of mammal species. In the current study, further insights into the interaction properties under constraints essential for the key interactions with the CAST4 based on the CAPN1 trace residues, were gained by analyzing the virtual alanine scanning mutation effects captured by the free energy difference of the protein stability and the binding affinity with the CAST4 inhibitor for either the CAPN1 itself or the CAPN1/CAST4 complex model structures. The targeting of key interaction residues for the CAPN1 against the CAST inhibitor was done for the purpose of identifying the aggregation characteristics and the pharmacophore factors as well as the geometry between the factors in the key interaction sites that can affect both the protease stability and activity.

## 2. Results and Discussion

On the basis of this complex model structure, it has been possible to explain these particular interaction preferences that may be the result of interaction with additional domains of the bovine CAPN1 by the trace residues (class or species-specific and conserved residues across mammalian CAPN group) in terms of a key residue’s point mutations. The mutant effects support the idea that the major specificity of the regulation system appears to be widely distributed across multiple domains along with the exposed surface groove, and not only around the catalytic center of Cys115 residue within the protease core. We embarked on a study that would predict the interaction effects of a single key substitution of a residue in bovine CAPN1 by evaluating the free energy difference based on changes in the enzyme itself and in the complex’s structure stability, in its of binding affinity with the CAST inhibitor, and its aggregation from the wild-type model structure. To investigate the importance of either the side chain or peptide bond of trace residues located on the interaction interface for the bovine CAPN1/CAST complex, we performed virtual alanine scanning by mutating each key residue to alanine.

### 2.1. Predicting Mutation Effects on the Relationship between Structure and Function of the Bovine CAPN1/CAST Complex Using Virtual Residue Scanning

In the first approach, this mutation was designed to predict the relative interaction preferences of the trace residues on the conserved inhibition motifs (residues 639–652 of LDDALDQLSDSLGQ motif of subdomain A, and residues 678–691 of KLGERDDTIPPKYQ motif of subdomain B) at each position within subdomains A and B of CAST4 to the overall CAPN1 binding affinity. In the same pocket, the mutated subdomain B of CAST4 had few conformer changes (from the wild-type form) due to this single mutation effect do little consider the conformational properties of the polypeptide backbone than the interaction of both side chains in the bovine CAPN1/CAST4 complex. However, regarding the mutation energy function, the conformer change derived from alanine scanning was reflected to a side chain and backbone entropy term of the polypeptide dependent on temperature.

#### 2.1.1. Predicting Mutation Effects on the KLGERDDTIPPKYQ Motif in Subdomain B of Bovine CAST4

**Figure 1:**
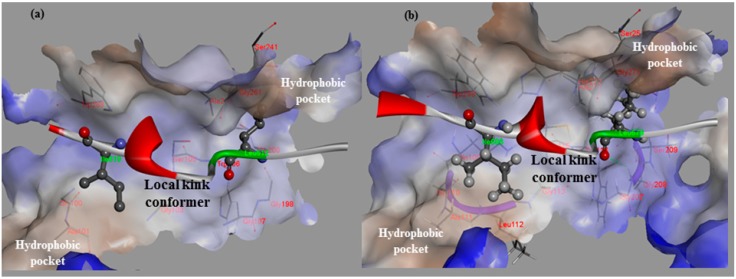
The hydrophobic interaction surfaces of the binding-site residues near the location at which the local kink conformer (residues 611–624 of rat CAST4 and 678–691 of bovine CAST4) binds. (**a**) Close-up views of the residues (Leu612 and Ile619) of CAST4 binding at the key contacts of rat CAPN2; and (**b**) the corresponding residues (Leu679 and Ile686) bound to the active site of bovine CAPN1. Both the subdomains B of CAST4 assume a similar backbone conformation for the distorted local kink. These local kink conformers show the most similar patterns of hydrophobic contact distribution across the CAPN subgroups. Hydrogen bonds are represented by green dashed lines.

**Figure 2:**
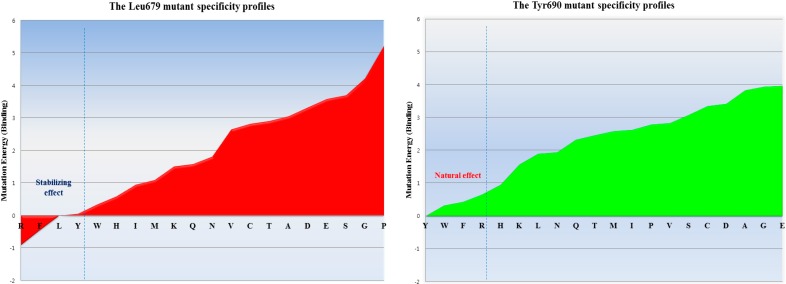
The inhibitor specificity of the residue preferences observed for each position in subdomain B of bovine CAST4. The Leu679 mutant shows preferences for the residues Arg, Phe, Leu, and Tyr (had stabilizing effects), whereas the residues Tyr, Trp, Phe, and Arg of the Tyr690 mutant had natural effects of the almost apparent wild-type complex (from most to least stabilizing). Those mutants show distinct preferences for residues bearing hydrophobic side chains (rather than hydrophobic ones) indicating specificity for both positions. In the single-point mutation study, the mutant conformer contributes considerably to the difference in mutation binding energy from the wild-type complexes. The energy difference of each mutation on binding affinity is the difference between the binding free energy in mutated and wild-type proteins, which are predicted to cause destabilizing effects such that mutation energies greater than 0.5 kcal/mol are designated as destabilizing.

Dramatic effects of these alanine replacements were observed; the Leu679 and Ile686 mutants of CAST4 (Leu679Ala and Ile686Ala of the subdomain B) permitted the peptide backbone to be more flexible and significantly reduced the hydrophobic interactions with the complementary binding sites (Cys115, Gly208, Gly271 and Ala273 of CAPN1). Each wild-type of the two key residues (Leu679, Ile686) was buried in two hydrophobic pockets (key hydrophobic contact residues; Gly271, Ala273 for Leu679 and Ala111, Leu112 for Ile686) to provide further stabilization via van der Waals contacts with the interface formed from domains DI and DII of the bovine CAPN1 enzyme (Figure 1b). Regarding interaction preferences, these mutants do not even show two H-bonding interactions with the backbone of two glycine residues (Gly208 and Gly271) since the distance between them is too far to form any H-bonding (exceed the distance threshold 2.5 Å), in contrast to the wild-type complex. Consequently, the two mutants (Leu679Ala and Ile686Ala of CAST4) would be unfavorable for binding bovine CAPN1, destabilizer than the wild-type complex as 3.03 kcal/mol and 2.22 kcal/mol respectively. The prediction further performs a search for stabilizing multiple mutations on a set of any of 20 amino acids for saturation mutagenesis to determine the specificity of residues found at the positions (the positions of 679 and 686 for CAST4). In the cases where combinations of simultaneous mutations are generated, the mutations with the lowest mutation energy at the position from the wild-type protein are scanned. Then, a novel residue that satisfies the positions is identified, highlighting the importance of the sites for its inhibitor binding. This is reflected in the key factors in the active, site-directed CAST4 from the specific locations as shown in Figure 2. There seems to be a requirement for the formation of H-bonds between them as well as, contribution to essential hydrophobic interactions in the region of the active site for protease inhibition. Moreover, as the general preference, greater freedom of movement of the side chain for large side chain (in an inhibition potency in the order of arginine > phenylalanine > leucine > tyrosine) is required at the 679 position, and the bovine CAPN1 may tolerate tryptophan or, histidine within the hydrophobic binding site as shown in Figure 2. The mutant specificity (Figure 2) was defined by the highest selectivity of binding affinity from stabilizing to destabilizing effects at this position, but the priority of these residues seems unlikely to be very selective as they are also accommodated by the hydrophobic pocket of another CAPN member, CAPN2 (Figure 1a). Interestingly, the residues (Leu679, Ile686) of CAST4 were positioned at the start and end points in a serial β-turn and kink construct, and their orientation in relation to each other was in the opposite direction for the wild-type. The predominant interactions are mediated by a complement to the distorted backbone conformation as a local kink of residues 679–686 directly compact interacting with the active core cleft of bovine CAPN1. For these specificity profiles of bovine CAST4, the frame of the local kink at the positions must be even more important if other three-dimensional structures (for example, an extended β-strand conformation consisting of proline peptide [36], which arise from nucleophilic attack by the thiol group of Cys115, preferably located Leu679 and Ile686 residues closer to the Cys115 residue, where it can easily undergo local folding prior to cleavage in a substrate-like manner. The binding mode of residues 679–686 points away from the enzyme active site and thus avoids cleavage that is apparent from the inhibitor capabilities (as shown in Figure 1).

On the other side of the binding mode, the Thr685 residue does not show stronger interaction with specific residues Gly271 and Trp298 than the main chain interactions between Gly680 and Gly271 residues in the bovine CAPN1/CAST4 complex owing to a relatively disordered and steric orientation to the rear of the protruding local kink. This was well documented by the alanine mutation effect when the Thr685Ala mutant was introduced by hydrophobic substitution from the polar group in its side chain; the mutation newly occurred due to hydrophobic contact with both Val269 and Trp298, in contrast to the Gly680Ala mutant, which was designed to abolish the electrostatic interaction with Gly113 and Cys115 of CAPN1. The entrance into the opposed chemical property of the variants (the Thr685 and Gly680 mutants to alanine) appeared that the forehand variant replenished for the backbone’s entropic penalty into less destabilizing effect (0.80 kcal/mol) than latter variant was most significantly reducing a relative of backbone flexibility (3.96 kcal/mol).

**Figure 3:**
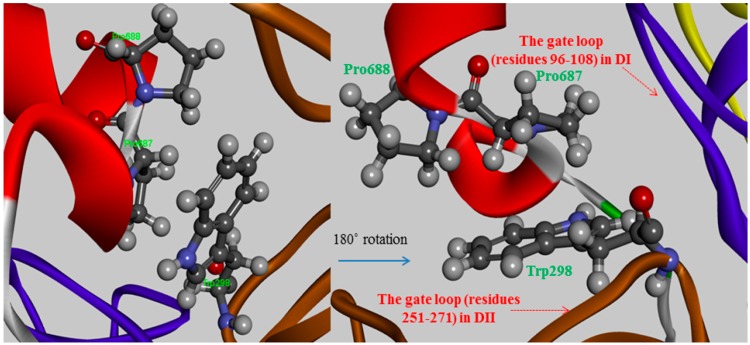
The π-π stacking interactions between two proline residues (Pro687 and Pro688) of CAST4 and a key tryptophan residue, Trp298 of CAPN1 from bovine in which Trp298 moved to tuck into a hydrophobic patch formed by the Ca^2+^-binding induced rearrangement of the gating loops (residues 96–108 in DI and 251–271 in DII).

The Pro687 and Pro688 mutants demonstrated that the π-π stacking interactions with the Trp298 residue play major roles in stabilizing their substructure and maximizing its binding affinity, the interaction strength of which depends on their relative positions. The Pro687mutant not settle down than the neighboring Pro688 mutant as a difference of greater than 3-fold magnitude as 2.12 kcal/mol and 0.66 kcal/mol for their mutants respectively. In comparing the two proline variants, the degree of destabilization effects between them could possibly originate from whether a parallel or diagonal π-π stacking interacts with the indole ring of the Trp298 residue via stacking conformation and stabilization effect for their wild-type. The side chain of Pro687 stacks in a coplanar conformation as a parallel π-stacking with respect to the indole group of Trp298, and it has a generate effect than that of the Pro688 mutant (Figure 3). Though these π-π stacking interactions are physically remote from the Ca^2+^-binding-induced to the active conformer, the alanine scanning effects at residues 687–688 do not account for this coplanar stacking into destabilizing the dipole moment of the Trp298 residue, or any non-polar contacts observed in the absence of those π-π stacking interactions. Therefore, if either of the relevant proline to alanine substitutions made no direct contact with the Trp298 residue that prevents access of the inhibitor to the active center with direct blockage of the catalytic residues, the alternation effects might interpose to some extent an additive hindrance to the mutated complex. Two proline residues (687–688) have a restrained backbone conformation, which likely contributes to maintaining an unstructured conformer of the inhibitor protein. These residues also served specificity profile for the positions is not cleaved unlike the substrate of the enzyme. The observed overall mutation effect using virtual alanine scanning for the conserved TIPP peptide the mediating the inhibitory activity of CAST4 showed good agreement with the results of earlier studies by Betts *et.al*. [37]. One major structural attribute in the TIPP sequence is likely to bind inhibitor, that is, the backbone disorder of the peptide after all.

**Figure 4:**
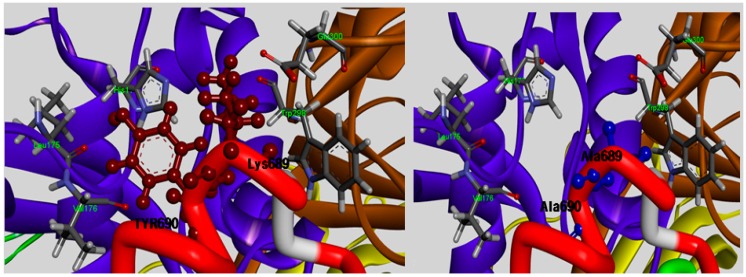
Detailed view of the interaction sites of the Lys689 and Tyr690 residues of subdomain B, which are in contact with the entryway of the protease core DI and DII (shown in ribbon represented in blue and brown, respectively) and were mutated to alanine to reduce the size of its side-chain, rendering CAST4 less space-filling mode from the active site.

The TLPPKYK sequence in CAST1 and TIPPDYR sequence in CAST3 can be found in the N-terminal of the conserved TIPPXYR motif, but they are not detected in CAST2 corresponding to the TIPPKYQ inhibitory motif within the subdomain B of bovine CAST4. The TIPP peptide (residues 685–688 of the bovine CAST4) resulted in no obvious difference between its inhibitory profile and those of other mammal CAST but not the KYQ peptide (residues 689–691) located in the two turn helix conserved across the species. The position of the KYQ peptide-bond makes its lining out of the activated pocket cleft, whereby it may indirectly affect CAST inhibitor activity through interactions with the flexible regions on both sides of the entrance of the protease core of CAPN1. The comparison of the binding affinity of both Lys689 and Tyr690 mutations to the wild-type, showed very interesting results in this study. The substitution of Tyr690 with alanine showed more than a 1.7-fold decrease from 2.29 to 3.38 kcal/mol in the CAPN1 binding, as shown in Figure 2, relative to the Lys689 modification (1.62 kcal/mol); these wild-type residues are bound to the enzyme surface in each position that corresponds to the localization of the exposed reactive site loop in the domains DII for Lys689 and DI for Tyr690 residue, and their mode of interaction with the protease binding loop is substantially different (Figure 4).

The Lys689 mutant causes its side chain to hold more rigidly in the hydrophilic contacts to considerably decrease electrostatic interactions with the polar loop region (Cys108, Gln109, His179, Trp298 and Glu300 residues) in DII, in particular to the primary amino group with a positively charged in Lys689 no longer enhances the electrostatic contact with side chain of Glu300 as a result of the replacement of the methyl group. More surprising was that the strongest stacking between Tyr690 and His179 in DI occurred in the presence of Trp298, an apparent consequence of the large surface area of their large side chain, and the dipole moments were devoid of change with alanine at the position. The absence of this staking in the Tyr690 mutant may alter a set of direct and indirect contacts that affect the positioning and orientation of the π-π stacking done by Trp298, which results in acting for the statically blocking access to the binding loop residue, and also electrostatic properties of both the His179 and Tyr690 residues. In contrast, the mutation of Tyr690, in which a methyl group was inserted between Leu175and Val176, the shielded the hydrophobic residues from the aqueous solvent but was insufficient alone. In particular, large amino acids, such as tryptophan or phenylalanine, at the position should contribute greatly to preference by CAPN1 interaction with the electron-delocalized π-system showed a relative preference in Figure 2. The Tyr690 residue, which shows more than twice the inhibition of Pro687 residue, implies other preferences to an affinity of CAPN for the flexible binding loop in DI, especially on the C-terminal side. The Tyr690 mutant resulted in a dramatic loss of binding affinity at the position. This suggests that simultaneous π-π stacking interactions, in the presence of Trp298 residue with active site assembly seem particularly important for the inactivation of bovine CAPN1 by CAST4. The data in Figure 2 suggests that the order of suitable fitting possesses a variety of key features, such as three-dimensional structure, and the physical and chemical properties within the binding pocket revealed some preferences at the 679 and 690 positions. The mutant of the same residue, arginine, which when compared with the mutant complexes from its wild-type has an unequal response to non-polar binding sites. Thus, it is important to characterize their similarities and differences in positions; there is a far greater stabilizing effect for the 679 (−0.9 kcal/mol in the Leu679Arg variant) than 690 (0.0 kcal/mol, without the mutation effect of the Tyr690Arg variant) position based on the free energy difference of the binding of two molecular partners due to single-point mutation of selected residues. The difference in the stabilizing effect for the arginine mutants (Leu679Arg, Tyr690Arg) according to their locations has been made from the backbone conformer. This appears to be more important in defining the preference profile within the subdomain B of bovine CAST4. Inhibition could occur at the positions more or less independently of the specific residues presently bound to the subsites as it fulfills the fundamental factor of being a distorted structure, whereas the scope of inhibition might be less affected by the primary residues. Though both structures are required to confirm the mode of action with the complex, this should provide interaction contacts to stabilize the bound form of CAPN1 as a crucial printing improving inhibitory activity. Notably, the tendency of binding preferences at those positions (the 679 and 690 positions) of CAPN1 appear to be more important in defining the most potent inhibitors of the enzyme share Arg-Trp moieties. This was also characterized by Cuerrier *et al.* [27]. When compared, even if two positions are not responsive to the P3 and P4 positions in the peptidomimetic inhibitors, if sufficient features similarly are observed there is some difference in the relative preferences toward CAPN1, possibly explained by the stabilizing contributions of the other domains (DIII, DIV of CAPN1, and subdomain A of CAST4) of the complex in our model system.

A further interesting point is that Gln691 is only bovine-species-specific and it was not preserved across the subdomain B of each of the four repeating domains of the protein CAST (lysine in CAST1, arginine in the CAST3 and glutamine in the CAST4 from bovine). However, the hydrophilic residue should be at the position at which it could possibly result in the adaptation of alanine, which would otherwise have a poor fit. The position of the Gln691 residue is not subject to direct contact with the matching domain DI of the CAPN1, but the aqueous environment where the mutation renders it apparent as a lesser extent of decreasing binding (0.02 kcal/mol) at the subsite compared with the wild-type protein including some impact on the specificity of its polar property of the side chain. The bovine CAST4 specificity of both the Lys689 and Gln691 residues towards bovine CAPN1 is a difference of more than ten-orders of a decreased affinity that remains between their mutants, which is highly dependent on these positions. On the other hand, the Tyr690 residue should only be made with its nonspecific-inhibitor caution by the conserved motif of the subdomain B on the C-terminus of the CAST4.

**Figure 5:**
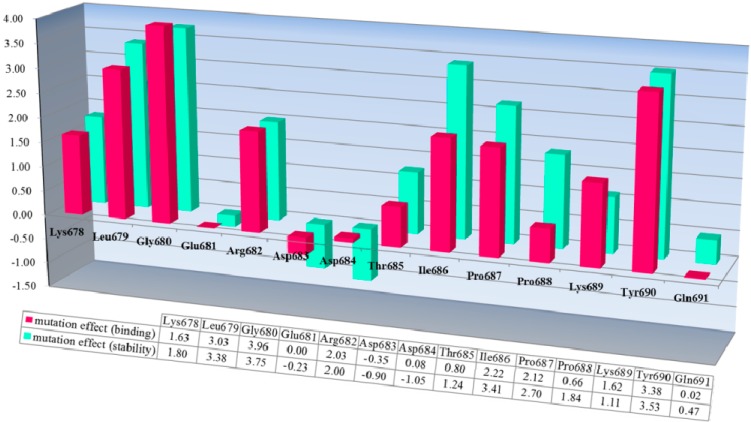
Inhibition profiles of its native inhibitor CAST4. The conserved KLGERDDTIPPKYQ motif in the bovine CAST4 (residues 678–691) was screened using a computational, alanine scanning mutagenesis. The results are shown as the residue preference from a wild-type complex with the corresponding energy effect (kcal/mol) of alanine mutation at the single-point position for the stability (green) and binding affinity (red) of the bovine CAPN1/CAST4 complex. Each bar chart of mutation energy is created to allow for comparing the different effects of mutation, when the mutation effects are defined as “stabilization” (mutation energy less than −0.5 kcal/mol), “natural” (mutation energy between −0.5 and 0.5 kcal/mol), and “destabilizing” (mutation energy greater than 0.5 kcal/mol).

The corresponding mutation effects (calculated energy effects) in terms of the stability of the bovine CAPN1/CAST4 complex structure (a green bar chart) and binding affinity (a red bar chart) with the bovine CAST4 are shown in Figure 5.

**Figure 6:**
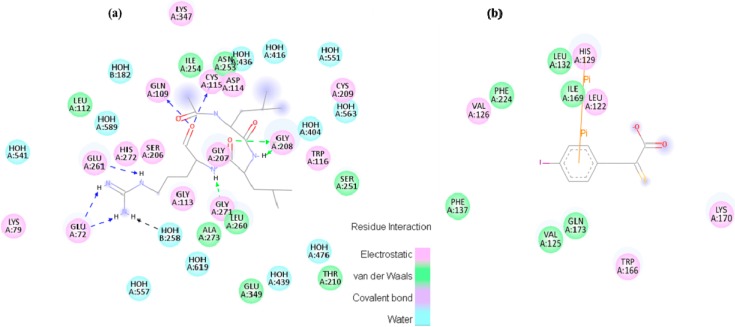
Small molecule inhibitor interaction diagrams for the binding pocket of the mammalian CAPN subunits (PDB code: 1TL9, 1NX3). For both inhibitor-bound structures, inhibitors are shown as sticks accompanied by key residue representation of 2D-CAPN interacting interface. The complex structures reveal the defining their physicochemical properties for CAPN selectivity and specificity: (**a**) In the complex structure between rat CAPN1 and leupeptin (1TL9) at the protease core, extensive interactions help stabilize leupeptin at the active site by eight hydrogen bonds with the side-chains of Glu72, Glu261, Gln109, Cys115 residues (a blue dashed arrow) and backbone of Gly208, Gly271 residues (a green dashed arrow) and hydrophobic interactions with Leu260, Ser251, Ala273, Asn253, Ile264 residues. The domain-leupeptin complex overlaps with those of the CAPN/CAST complex structure (PDB code 3DF0) as main chain atoms r.m.s.d of 1.4 Å [38]; (**b**) The interaction site for the crystal complex structure (1NX3) of CAPN domain DVI (of pig) and its inhibitor PD150606 bond in a hydrophobic pocket (Val125, Leu132, Phe137, Ile169, Gln173, Phe224) of DVI where it makes favorable π-π interaction with the side-chain of His129.

Both mutation effects (stability and binding affinity) displayed analogical inhibitory preference results that generally show almost identical relative CAST4 mutation effects having a correlative tendency (the correlation coefficient is observed by 0.92) toward bovine CAPN1, consistent with their binding site. Interestingly, the differences between the effects of the Asp 683 and 684 mutations to alanine at the positions for stability and binding affinity suggest that the other domains in full-length have a greater impact on the complex stability than binding affinity related to the inhibition specificity. These positions are far enough from the hydrophobic sites that they must be influenced by the local kink construct. Conversely, the general preference for bulk hydrophobic residues, Ile, Leu, and Tyr (at positions 679, 686, and 690) is consistent with fitting into the equivalent large hydrophobic pockets within the protease core of bovine CAPN1, where their inhibitory efficiency is similar to that of the other CAPN2 subgroup. Indeed, the Gly680Ala mutant suggests that this β-turn backbone portion of the inhibitor is highly flexible when complex with the protease core to avoid its steric packing (Figure 1 and Figure 5). However, additional inhibitory preferences were observed with the KYQ peptide (residues 689–691) of bovine-specific residues. The unfixed sequence (KYQ in the conserved TIPPXYR motif) influences the selectivity of bovine CAST4 at these positions, but it does not need to be similar to optimized inhibitor candidates, because the leading structure may orient to an extent sufficient to direct the enzyme inhibition independent of whether the primary sequence is adequate for the active site. Consistent with the central roles of the residues at the binding pocket, active site-directed inhibitors [26,27,28,29,37] block their susceptibility to CAPN1 similar to the CAST4 preference in the corresponding positions (Figure 6) throughout the same non-covalent interactions found in the rat, humans and our bovine model. The generating specificity of both active site-directed inhibitors is illustrated in Figure 6.

The major interactions, during which H-bonding and hydrophobic interactions between active site residues and the inhibitors serve to stabilize the complex during Ca^2+^-binding and to orient the inhibitor as a common feature, and other marked deviations from the structural characteristic are also observable in both complex structures. In the immediate vicinity of peptidomimetic inhibitors, the secondary structure, on the other hand, behaves exclusively as a major obstacle where CAPN attack occurs since these portions of the inhibitors are highly flexible when competitively bound in the active site to the enzyme substrates. The β-strand conformation (PDB code 1TL9 and 1TL0) is apparent from these specificity profiles for protease recognition as one of the major factors that is not considered when deciding whether to develop reversible or irreversible inhibitors. The conformation accelerates additional H-bonding and an antiparallel β*-*sheet of both backbones with the key residues (the Gly271, Gly208, Cys115 residues), and then the ability of the peptide to inhibit CAPN is altered. This further makes bovine CAPN1 a target for the computationally aided molecular design of novel active site-directed inhibitors. A potent inhibitor specific for bovine CAPN1 could be designed to scrutinize allosteric or other binding sites such as domain DIV, which when ligated by CAST4 imitation, renders the enzymes unable to achieve their proteolysis function. It must be carefully considered whether the targeting interaction sites would be subject to either autolysis or subunit dissociation or aggregation.

#### 2.1.2. Predicting Mutation Effects on the Two LGMD2A-Accociated Mutations (R385H and D600G in the Corresponding Positions in the Bovine CAPN1)

**Figure 7:**
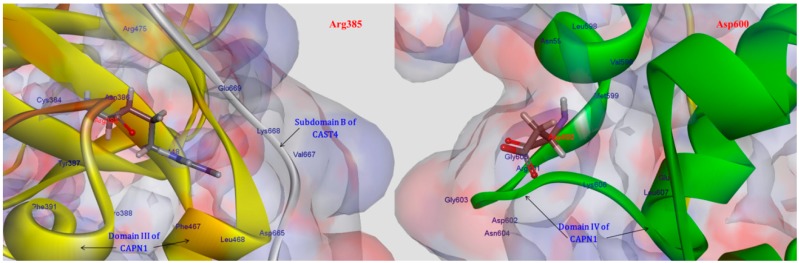
Interaction residues associated with two LGMD2A-related mutants (Arg385His, Asp600Gly).

More importantly, additional tendencies of preferences were observed with the subsite of an exposed bent loop (residues 663–669) on the N-terminus of subdomain B in CAST4, which is adjacent to the interface of the Arg385 residue related to the LGMD-2A mutation’s position (R448H in CAPN3). These tendencies were not predicted resulting in a distinct difference of specificity from previous studies on the inhibitor selectivity of CAPN1 [34]. We were interested in two LGMD2A-associated mutants, R448H and D705G, which retain the proteolysis activity of either of these CAPN isoforms (CAPN3, CAPN2); however, they significantly affect the stability of the protein itself and are also diseases causing [8]. In the current study, of known pathogenic mutations in CAPN3, it was possible to predict the effects of two mutants for pathogenic missense mutations (R448H and D705G) in LGMD2A in terms of the bovine CAPN1 inactivation and its complex stability through the CAPN1/CAST4 complex model. We made an assumption based on a sequence homology (more than 45%) that the structure should be similar to CAPN1 and should share biochemical properties, such as Ca^2+^-dependent activation (in the nanomolar range of Ca^2+^ ions concentration) and maximal activity at natural pH, despite the presence of three exclusive sequence inserts (NS at the N-terminus, IS1 in the domain DII, and IS2 between domain DII and DIII) without small subunits [7]. Both mutants R448H and D705G in CAPN3 should be projected onto R385H and D600G in the corresponding positions in the bovine CAPN1. The Arg385 residue is situated on a loop of domain DII that makes intramolecular domain contacts (DII/DIII) and intermolecular interfaces (CAPN1/CAST4) on either side of the center of the loop, and it may leaven the assembly and activation of the enzyme. On the contrary, the Asp600 residue is located elsewhere in the EF-hand2 motif (residues 587–620) of domain DIV; it is exposed to solvent in both the absence and presence of Ca^2+^ ions and has no interactions with reactive residues of the CAST4 (Figure 7). Surprisingly, the Asp600Gly mutant adjoins to the dimerized interfaces (domains DIV/DVI) between CAPN1 molecules through the fifth-EF-hand motif such that its stability must be dominated by the heterodimer formed.

One possibility, we considered was that the Arga385His mutant could affect stabilizing, electrostatic interactions (salt-bridge) with the enzyme itself and some binding partner, CAST4, in such a way that it altered the coupling strengths of partners (the residues Asp665, Val667, Lys668, Glu669 on N-terminus of subdomain B) interacting with Arg385 residue in both interaction regions (DII/DIII) and that it could play an important transfer part in Ca^2+^-induced activation signaling. When we analyzed the complex model, the residues Asp665 and Glu669 located in the bent loop of CAST4 were considered potential partners for salt-bridge formation according to the strength governed by their positions on the flexible loop. As another factor, both residues of subdomain B are also localized at the external surface of the CAST4 across the domain DIII of CAPN1 as seen in Figure 7. The mutation of arginine to histidine at the 385 position could decrease electrostatic interactions with the adjacent hydrophilic residues within both the CAPN1 and N-terminus subdomain B of CAST4 originating from its basic property and charge variation. In particular, the internal salt-bridge would finally result in a much greater reduction in the structural stability of the complex (2.70 kcal/mol) than of the enzyme itself (0.20 kcal/mol) in relation to both their wild-types (Table 1).

The Arg385 residue is distant enough from the protease core that it should retain the proteolysis function of the enzyme, but the mutant considerably affects fissure of the CAPN1 structural integrity in the presence of the inhibitor CAST4. The structural stability was much lower in the unbound state than in the bound state, that is, almost 14-times lower. Moreover, the substitution did not directly change the enzyme folding but significantly disturbed the tighter association with subdomain B since, at the scanned position, which is not positioned and oriented at an optimal distance for the salt-bridge-interaction of acidic partners (the residues Asp665 and Glu669 of CAST4), so the wild-type salt-bridge is no longer available as a result of the Arg385His mutation. Correspondingly, the basic loop flexibility allows a small increase in the entropy of the complex, as if the conformer change of the local site caused by the mutation is not sufficient to influence for the Ca^2+^ion-binding of either the inactive or active enzyme form to any significant degree (mutation energy effect of −0.23 kcal/mol as neutral effect). However, in the case of the CAPN1/CAST4 complex, the reduction of electrostatic interactions (salt-bridge) with the inhibitor was more unstable in the bound state than in the unbound state (with increased binding free energy of 1.85 kcal/mol from that of the wild-type complex as a destabilizing effect) and could let down the binding affinity of CAST4. Thus the variation at the 385 position has a larger impact on the inhibition of CAST4 via the structural stability of the complex than the efficiency of association between them.

In contrast to the Arg385His mutant, it is feasible that the Asp600Gly variation would have a smaller impact on structural stabilization and would behave similarly in the absence or presence of protease inhibitor dominated by its ability to form a heterodimer. The stability of the variant at the 600 position was shown to be greater in its inhibitor complex (−0.99 kcal/mol) than in the enzyme itself (−0.55 kcal/mol) against either wild-type.

**Table 1:** Evaluating effect of single-point mutations on stability and binding affinity.

	CAPN1 Arg385His	CAPN1 Asp600Gly	CAST4 Leu643Ala	CAST4 Leu646Ala	CAST4 Leu650Ala
^†^ Mutation Energy (the enzyme itself stability)	0.20 kcal/mol	−0.55 kcal/mol	-	-	-
* Stability effect	Neutral	Stabilizing			
^†^ Mutation Energy (the complex stability)	2.70 kcal/mol	−0.99 kcal/mol	1.70 kcal/mol	1.96 kcal/mol	2.09 kcal/mol
* Stability effect	Destabilizing	Stabilizing	Destabilizing	Destabilizing	Destabilizing
^‡^ Mutation Energy (Ca^2+^-binding)	−0.23 kcal/mol	−0.99 kcal/mol	-	-	-
* Ca^2+^-binding effect	Neutral	Stabilizing	-	-	-
^‡^ Mutation Energy (CAST4-binding)	1.85 kcal/mol	−0.43 kcal/mol	2.39 kcal/mol	2.88 kcal/mol	2.50 kcal/mol
* CAST4-binding effect	Destabilizing	Neutral	Destabilizing	Destabilizing	Destabilizing

^†^: Structural stability is determined by the difference between the folding free energy of mutated structures and the wild-type enzyme for the single-point mutations; ^‡^: Binding free energy is defined as the difference between the free energy of the complex and unbound state; *: Mutation effect is defined as follows that stabilization indicates that the mutation energy is less than −0.5 kcal/mol, neutral is mutation energy between −0.5 and 0.5 kcal/mol, and mutation energies greater than 0.5 kcal/mol are designated as destabilizing.

The stability gain of the Asp600Gly mutant that was observed on heterodimerization with engaging the major hydrophobic interface that holds together through the pairing of their EF-hands motif of between CAPN1 molecules and not only that bound in a direction opposite to an amphipathic α-helices of the subdomain A of its inhibitor CAST4. The structural stability would be maintained by the CAPN1-induced helical anchors of subdomain A. After mutation, the structural stability became too high for effective protection through the hydrophobic residue’s packing form in an aqueous environment. Thus, the mutation energy for CAST4 binding was lower (−0.43 kcal/mol) than that for Ca^2+^-binding (0.44 kcal/mol) for more increasing binding affinity with the former Arg385His. However, this would have a relatively small impact on the mutation energies, which were in the neutral range from −0.5 to 0.5 kcal/mol, which mean that the mutation had a very small effect on enzyme function. The variation of any acidic residue at the 600 position is required for the Ca^2+^-chelating residues. As summarized in Table 1, two variants (Areg385His, Asp600Gly) showed differences between the folding free energy of mutated structures and binding free energy with molecular partners (Ca^2+^ ions and CAST4) in their mutated complex structures. The variants allowed CAPN1 to hold its active conformation within the Ca^2+^-induced structure, but the preference for inhibition of CAST4 was different for each mutant; the Arg385His variation significantly decreased its binding affinity to CAST4 due to its complex’s instability.

On the other hand, the Asp600Gly variant was less efficient in forming the complex than a heterodimer with the other CAPN; it had a stabilizing effect on the complex but did not affect cohesion with the inhibitor. In addition, the Arg385His mutant contributed to revealing the important interaction sites (the bent loop of residues 663–669) on the N-terminus of subdomain B of CAST4 for enzyme regulation. Furthermore, the importance of the bent loop for the CAPN1-CAST4 binding was previously unknown.

#### 2.1.3. Predicting Mutation Effects on the LDDALDQLSDSLGQ Motif in Subdomain A of Bovine CAST4

**Figure 8:**
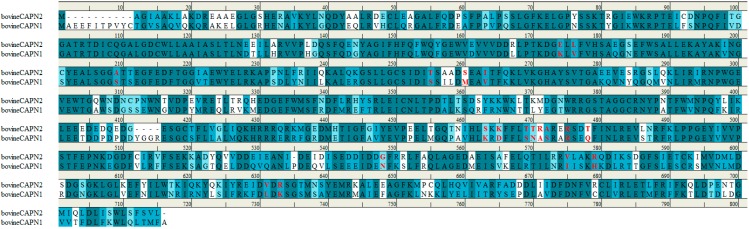
Sequence alignment of bovine CAPN1 and CAPN2 large catalytic subunit. The accession numbers for these sequences are NP_776684.1 (NCBI reference sequence) and AAI34527.1 (GenBank), respectively. Bovine CAPN1 has 81.5% sequence homology to the subunit of CAPN2. The trace residues that made up the class-specificity of CAPN in the interaction sites with subdomains A and B of the CAST4 group are indicated in red. All positions are described in terms of the bovine CAPN1 amino acid sequence. For comparison, two bovine CAPN isoforms had similar specificity toward the enzyme CAST4.

**Figure 9:**
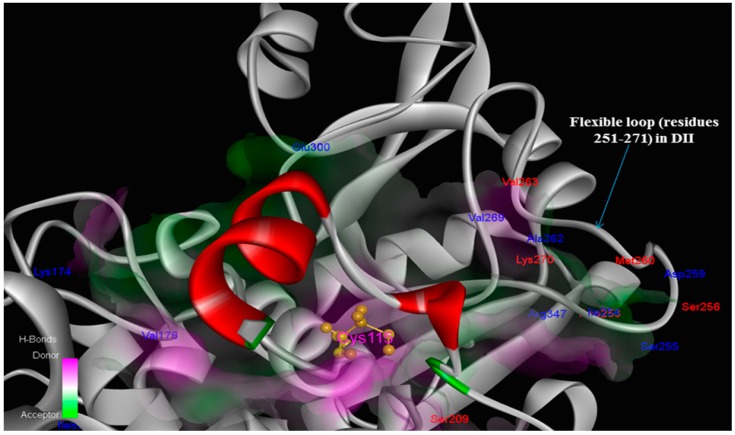
Interaction sites within the protease core by residue character: class-specific (blue; Ile83, Lys174, Val176, Ile254, Ser255, Asp259, Ala262, Val269, Glu300, Arg347) and bovine only species-specific (red; Ser209, Asp253, Ser256, Met260, Val263, Lys270) trace residues among key interaction contacts made the CAPN1/CAST4 complex. Notably, not adjacent regions of the catalytic Cys115 residue, the positions of trace residues from bovine CAPN1 are location-focused within the open conformation of the flexible loop of domain DII (residues 251–271) which gates active site, consistent with their binding site of conserved KLGERDDTIPPXYX motif of CAST4 subdomain B having similar inhibitory preferences in the active site cleft, but will likely not be identical against two CAPN isoforms (CAPN1 and CAPN2) from the bovine.

Subdomain A of CAST4 bound to a hydrophobic pocket formed by EF-hands one (the residues 543–578) and two (the residues 587–620) in the domain DIV filled by the inhibitor. Peculiarly, the conserved hydrophobic Leu residues 643, 646, and 650 inside an amphipathic helix (the residues 638–650) of subdomain A were buried deep in the allowed binding region, which helped to form strong hydrophobic interactions with the domain DIV. Several bulky residues (Leu552, Leu556, Phe612, Trp616, and Leu623 of DIV) in the hydrophobic pocket closely embrace the Leu residues to form favorable contacts with between side chains. The breadth of the hydrophobic groove seems like an opened baseball glove, within which the inhibitor subdomain A is somewhat flexible. The conservative interaction residues, both of them in the bovine complex, were further condensed as shown in Figure 8 and Figure 9. Even a single site mutation in either region could have an effect on the affinity of either region alone. This can be seen by estimating the SAP (Spatial Aggregation Propensity) whereby inhibition can occur as a complement to the subdomain A of CAST4, directly interacting with the key trace residues (colored blue and red) on the exposed hydrophobic region as shown in Figure 9. While the Leu residues (Leu643, Leu646, and Leu650 in the residues 639–652 of LDDALDQLSDSLGQ motif of subdomain A) have always been thought to be embedded in the inhibition region, virtual alanine scanning (mutated to be less hydrophobic) was performed of either a single or multiple displacement to elicit an induced effect (for the complex’s stability and binding affinity) caused by the positional changes along with the hydrophobic interaction. The effect of each mutation of the three Leu residues (Leu643, Leu646, and Leu650) individually on alanine had varying results (Table 1). The stability of the complex was greatly reduced, even further than the binding capacity to the enzyme in each variation, illustrating the sensitivity of this region to changes in the hydrophobic interactions. These mutants were expected to display a reducing flexibility of the side chain and van der Waals contacts with the companion residues (Leu552, Leu556, Phe612, Trp616, and Leu623 of DIV) in the wider bounding pocket, thereby showing weaker preferences for the positions. The effects of these mutations on the hydrophobic pocket of the enzyme could be directly predicted by multiple mutations of a set of the Leu residues for the complex’s stability, when double mutations are generated from the retained single mutations and then the triple mutations are generated from both the former mutations (the retained single mutations and double mutations). Their multiple displacements to alanine show a sharp stability drop in a mutually dependent manner to approximately an order of magnitude as a high order of repeated variations (single Leu646Ala to double Leu650Alal and Leu646Ala to triple Leu643Ala, Leu646Ala and Leu650Ala mutants leads to an increase in the folding free energy from the wild-type complex with values of 2.88, 5.21, and 7.11 kcal/mol respectively. The data is not shown in Table 1) with a negative effect on the inhibition. The increase in the structural instability of these mutant complexes contributes to subdomain A binding to the hydrophobic pocket of the CAPN1 in the stabilization of the wild-type complex structure, resulting in stronger inhibition than that seen with the mutant complexes.

### 2.2. Targeting More Specific Site to Increase the Bovine CAPN1/CAST4 Complex Stability Using Hydrophobicity Exposed to Aqueous Solvent of the CAPN1 Residues (SAP Values for the Residues)

**Figure 10:**
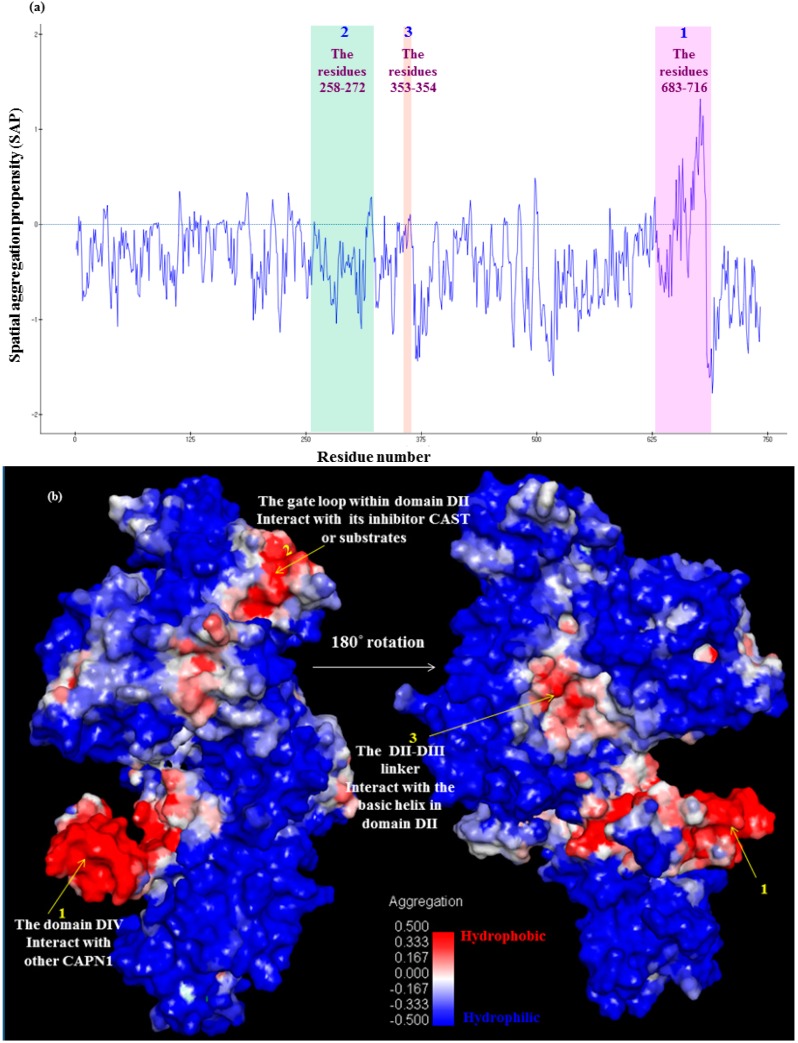
Spatial aggregation propensity (SAP) for bovine CAPN1: (**a**) Values of SAP at R = 10 Å which define SAP based atoms within radius-10 Å from a given atom for the catalytic subunit (domains DI-DIV) of active bovine CAPN1, along with peaks of chosen regions of the enzyme known to interact with inter-domains or other proteins; (**b**) SAP values (R = 10 Å) mapped onto the active bovine CAPN1 model. Positive SAP values are red (hydrophobic) whereas negative values are blue (hydrophilic); therefore, a highly exposed hydrophobic fragment would be deep red and a highly exposed hydrophilic fragment would be deep blue.

Molecular simulations using SAP were used to predict the bovine CAPN1 aggregation prone regions, which will be the subject for screening variants for which enzyme stability can be increased without losing its CAST4 binding affinity. Furthermore, enzyme stability is crucial when considering diseases associated with its destabilization. Protein aggregation is directly responsible for several diseases, such as type II diabetes and Alzheimer’s. One of the severe consequences of CAPN pathological activation is that over-activity of the enzyme frequently exacerbates many degenerative diseases [11,39,40]. For protein aggregation, hydrophobic interaction was shown to be the predominant interaction and also an important driving force behind binding at the interface of the heterodimer of CAPN1. We analyzed the bovine CAPN1 aggregation propensity, which is a measure of the tendency of surface residues to be aggregated among the trace residues exposed in solvent. The sites on the enzyme surface with high aggregation propensity scores specify regions that are prone to aggregation; there should be a more extreme intrinsic aggregation tendency of CAPN in such domains as DIV/DVI for the hydrophobic interfaces exposed to aqueous environment retained before and after Ca^2+^-binding. The variability of the increased tendency to enzyme aggregation will also depend on how many aggregation regions are present therein and on the effect of mutations on the stability and activity of CAPN1. Destabilizing mutations of the enzyme/inhibitor complex’s stability will shift the reversible equilibrium toward the unbound state in the complex system and can lead to a decrease in the inhibitory activity of CAST4. We observe that this was confined to a few regions with positive peaks for SAP, indicating high exposed hydrophobicity (Figure 10a). The enzyme-aggregation-prone regions (red on the SAP map) clearly indicate that the highly hydrophobic regions within the activated bovine CAPN1 are located on the functional interfaces. Two prone regions in hydrophobic patches 2–3, as seen in Figure 10b, disclosed intradomain conformational changes of domain DII on Ca^2+^-binding in which opening of the active site cleft occurs as the gating loop (251–271) and movement of the domain DII-DIII linker relative to the basic helix in domain DII to produce a more assembling to the catalytically active conformation. Hydrophobic patch 2 (residues 258–272) among the prone regions, takes in the active catalytic site, in particular His272; it provides, access of the bulkier inhibitor to a wider active site gated by the flexible loop (the residues 251–271). The loop in domain DII is variable across the mammalian CAPN group, providing potential for inhibitor selectivity (as the trace residues Ala262, Val263, Phe265 and Lys270 exist in the prone region) by defining the width of the protease core cleft on the hydrophobic patch 2 exposed. When His272 is converted to alanine, the mutant shows a lack of activity but an increase in the hydrophobic environment in Ca^2+^-dependent proteolysis and aggregation, respectively. The His272Ala mutant seems to be unable to instigate the nucleophile reactivity of the thiol group of Cys115 as observed previously in the His262Ala of CAPN2 [41]. However, in the complex formation with its inhibitor, this attribute of the coverage of patch 2 on the flexible gating loop (the residues 251–271) by the subdomain B of CAST4 is protected from aggregation throughout them shielded from water molecules. Hydrophobic patch 2, therefore, plays a crucial role in both the active conformation and function of bovine CAPN1 for stability of the complex with its inhibitor in the presence of Ca^2+^ ions. In the Ca^2+^-activated CAPN1 enzyme, the domains DIV and DVI also show interdomain conformational changes, and the catalytic hydrophobic core becomes accessible to CAST4 (at the subdomains A and C), leading to the enzyme/inhibitor complex where hydrophobic dimerization surfaces are positioned in aggregation prone region 1 (Figure 10b). This appears to prevent CAPN aggregation by non-covalent binding to both DIV and DVI coincidentally as if the following small subunit’s dissociation may be a major factor responsible for the Ca^2+^-induced aggregation of CAPN1. More interesting, however, are the residues (Phe690, Phe704, Phe707, Gln711, Leu712 and Thr713) that were not located at dimerization interface (but Met687, Met714, and Phe715 are highly conserved at the dimerization interface across the mammalian CAPN group) with other CAPN1 molecules among the bovine-specific trace residues within hydrophobic patch 1 with high SAP values (Figure 10a).

**Figure 11:**
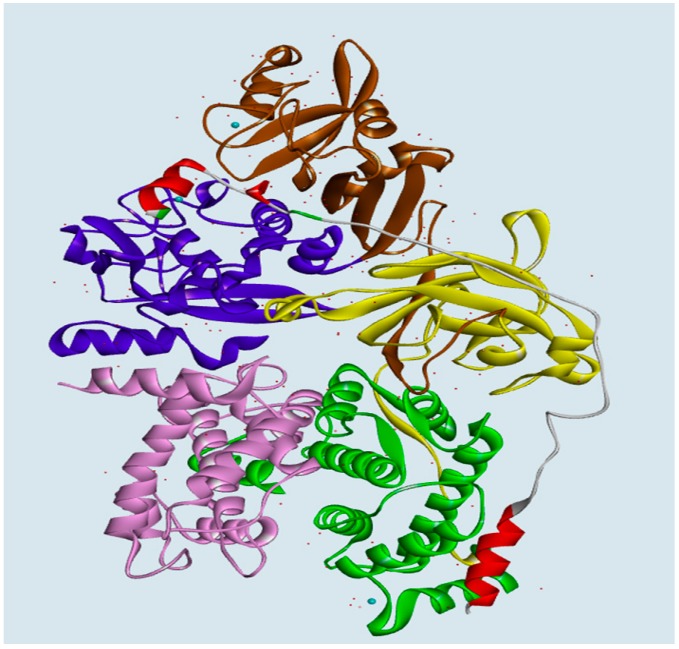
Overview of the 3D-complex model structure of bovine CAPN1 bound to CAST4. The modeled structures of the bovine CAPN1/CAST4 complex all have the same domain color schemes in the presence of Ca^2+^ ions: DI (blue, residues 30–220), DII (brown, residues 221–386), DIII (yellow, residues 387–543), DIV (green, residues 544–716) in the catalytic subunit of bovine CAPN when the CAST subdomains A–B and the three Ca^2+^ ions are shown as ribbon diagram and sky blue spheres, respectively. The domain DVI of small subunit (colored in pink) had not been characterized by our homology modeling and has been proposed as a homo-dimer model of the CAPN1 using the crystal structure of CAPN domain VI in PDB code 1DVI (Blanchard *et al.* [42]).

These residues show that a much greater selection of residues is expected to fit to the complementary EF-hands in DVI, allowing EF-hands4 (682–716) of domain DIV to activate the extensive hydrophobic contacts into additional stabilization, and then to affect its variable plasticity in the heterodimer. Thus the residues are positioned in a direction opposite to binding subdomain A of CAST4 that does not seem to be directly seen through their inhibitor selectivity, which must be achieved somewhere else in the non-aggregation prone region such as the hydrophobic core (the interaction region between subdomain A of CAST4 and CAPN1 in Figure 11).

This probably has an influence on subdomains A and C of CAST4 by enabling the hydrophobic cores of CAPN to be instantaneously anchored in domains DIV and DVI and inducing tighter cross-binding between subdomain B of CAST4 and domains DI-DIII via increasing inhibitor efficiency. Three prone region’s destabilization may lead to aggressive intramolecular domain interactions and may impair the assembly and activation of the enzyme. This being caused by such an exposure is energetically unfavorable and even more disfavored in an aqueous environment as it would accelerate random association between them leading to aggregation. Accordingly the CAPN that did not assemble into either its active form or into heterodimers might be resistant to CAST inhibition but it could be subject to aggregation in the Ca^2+^-dependent. The active enzyme is rapidly subject to auto-proteolysis, and subunit dissociation is better at high Ca^2+^ ions-concentrations. As revealed by the present study, the correlation between the highest SAP region and the known aggregation region is so high that the SAP values in the prone region can be the enzyme’s descriptor for the structure-based design of a CAPN-specific small inhibitor, such as PD150606 [31] in Figure 6b to extend the overall efficiency of the inhibitor candidates against over-activating CAPN via SAP stabilization. These aggregation prone regions (patches 1–3) where biochemical constraints were conserved in the CAPN1 do not modified object, but other critical flanking residues should increase the enzyme’s stability that radically shifts in the class-specific and the bovine species-specific residue positioned away from the interaction interface with CAST4 therein.

Information about the importance of hydrophobic interactions and the effects of CAST4 gained from the high SAP regions (patches 1–3), can providing significant new insights into aggregation that can be useful in screening more specific target sites to stabilize both unstable SAP regions and the complex stability with CAST4 at once. From all of the data obtained, CAST4 probably recognize overall activate CAPN1’s 3D-structural elements in the crude enzyme but absent from one part of fragments (the protease core in domains DI and DII) with the inhibitor’s interaction determinants. More distant interaction preferences of the bovine regulation system were predominantly distributed in the class-specific and species-specific residues for enzyme activation and inhibition by CAST4. In the case of subdomain B of CAST4, the preferences at key positions moved towards the *N*-terminus; hence, our data indicate its importance. Alanine mutations in the *N*-terminal part of subdomain B have a greater specificity effect on CAPN1 binding than do mutations in the C-terminal part for CAST4 inhibition. To advance our understanding of the inhibition determinants, we found a particular correlation with hydrophobicity exposed to solvent (SAP values for residues) for the complex’s stability and the binding affinity of both of them; in particular, the stabilization of CAPN1’s aggregation prone regions improved to inhibitory activity of CAST4. These findings should not be ignored in the design of small inhibitors having selectivity for specific CAPN isoforms.

### 2.3. Protein-Protein Interface Determinant Residues Using a Combination of Alanine-Scanning Mutagenesis and Receptor-Based Pharmacophore Modeling

As previously mentioned, most interaction interfaces between bovine CAPN1 and CAST4 are high SAP regions. This study on the aggregation prone regions of CAPN1 has shown that hydrophobic patches contribute greatly to the complex stability and inhibitory preferences between the proteins. Hydrophobicity is a leading force in protein-protein interactions, and the number of patches, in which may vary from 1 to 15, and their size may range from 200 to 400 Å cause an increase in entropy in stable complex formation. Most of the movements within these interfaces are governed by side chains and perturbations of local loops both from polar aqueous to nonpolar environment, resulting in the expulsion of water molecules in the hydrophobic interface. Protein-protein interactions have been frequently observed on much larger nonpolar surfaces and flatter interfaces (such as proteases interaction interfaces as 2000–4660 Å2, especially, the CAST4 buried approximately 2800 Å2 of the interface of CAPN2 in the crystal structure, 3BOW [43]) than the protein-small molecule contact surface, so that cannot be easily blocked by small inhibitor molecules for proteases. However, most of the protein-protein binding affinity is related to few key residues at intermolecular protein interfaces; hence, the exchange of residues critical for the affinity of interactions may almost abolish protein-protein binding. Identifying these key residues would aid in the rational design of complexes with high affinity and specificity as well as that of small inhibitors that can mimic the structurally conserved epitopes of the protease complex system. Alanine scanning mutagenesis enable the detection of the structurally conserved motif of which, on average, 79% of the key residues are located on complementary pockets, and 93% of the residues with a free energy difference of binding ( $\text{∆∆}G_{\left(mut\right)}^{binding}$ ) higher than 4 kcal/mol was found [44]. Nevertheless, in each case, residues whose mutation results in such a large distinction are unusually dependent on the complex characteristics, such as activity, binding partner, stability, or aggregation to compensate these negative effects. In particular, in the protease system, the threshold had to be lowered to 1.5 kcal/mol to obtain enough significant data. The mutation effect for some, but not all of active site residues is equipoise between activity and stability, as if a mutation that increases the catalytic activity is more likely to be destabilizing. In the opposite case, if a mutation increases the stability of a protein the catalytic activity will be decreased [45]. We suggest that local instability of the CAPN1 enzyme may be necessary for substrate binding and proteolysis by equipoise to expropriate three prone regions as shown in Figure 10.

**Figure 12:**
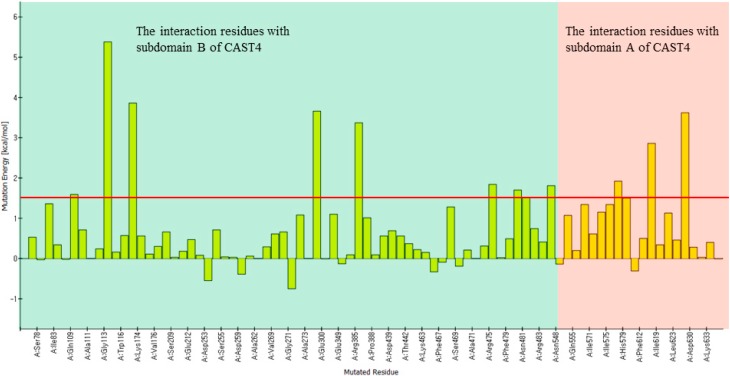
Contribution of only interaction interface residues to net binding energy in the CAPN1/CAST4 complex. Difference in binding free energy between alanine-substituted and wild-type of the bovine CAPN1 ( $\text{∆∆}G_{\left(mut\right)}^{binding})$ at contact residues is represented by chart bars. In the interfaces, the majority of residues of bovine CAPN1 are predicted to cause destabilizing effects (mutation energy greater than 0.5 kcal/mol is designated as destabilizing), while negative values indicate that the binding affinity increased when the side-chain was substituted by alanine.

The current study may also provide the opportunity to interpret the high SAP roles of protein-protein interfaces (as the aggregation prone region of patches 2 in Figure 10, as well as mobile gating loops within the active site of DII concentrated in the trace residues (whether class- and species-specific or conserved) of the bovine CAPN1. Well conserved trace residues across mammalian within the interface are expected to be crucial for the function of the CAPN group. They can tolerate some outside variation, but overall, the physicochemical properties of the residue are conserved. On the contrary, a great variety of class- or species-specific trace residues can be conservation shifting sites between CAPN subgroups (CAPN1 or CAPN2); they represent a distraction of constraints for such functional divergence, indicating less important positions than the former for the original function. We performed alanine scanning mutagenesis to identify the few key residues involved in the interactions of CAPN1 with CAST4, and then derived pharmacophore models for the protein-protein complex towards understanding the interaction between an enzyme and its natural inhibitor. In the 20-modeled bovine CAPN1/CAST4 complex structures, the energy effect of each alanine mutation on the binding affinity were calculated on all of the residues of CAPN1 and the residues of CAST4 within 5 Å defined in a group of interaction interfaces. The calculated values of mutation energy for any one of the mutated residues and their residue type are shown Figure 12. In 83 interfacial residues, Gln109, Gly113, Lys171, Trp298, Arg385, Arg475, Ile480, and ASN481 from the bovine CAPN1 on the protease core with subdomain B of the CAST4, on the other hand, the residues of Lys578, His579, Trp616, and Arg627 on the hydrophobic pocket of DIV with the subdomain A, have mutation energies above 1.5 kcal/mol. This can be used as a reasonable cut-off for significant residues which may contribute to the binding affinity of the protease-natural inhibitor complex. The key residues for interaction with subdomain B can essentially be divided into two groups; the Gln109, Gly113, Lys171, Trp298 residues are far more conserved if located at a complemented pocket as the protease core than the Arg385, Arg475, Ile480, Asn481 residues if located within the rest of the interface toward a rim of between interdomains. As expected, highly conserved core sites at the interfaces are likely to be good candidates to target with a small inhibitor.

#### 2.3.1. The Definition of Binding Site (Consisting of Gln109, Gly113, Lys171 and Trp298) Constraints for Virtual Small Inhibitor Screening

**Figure 13:**
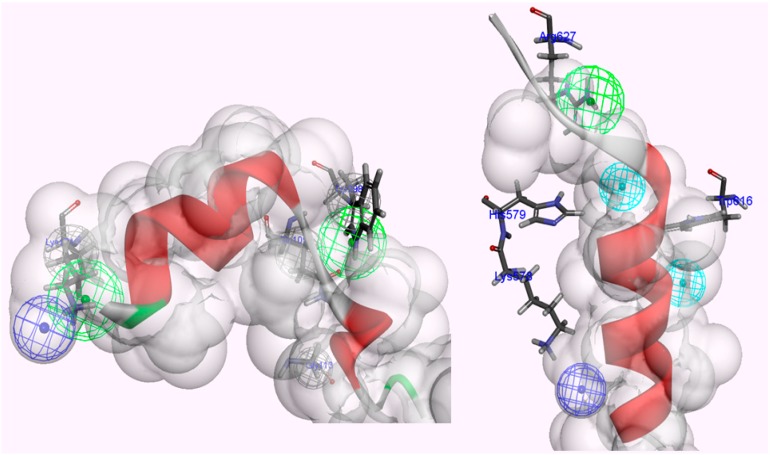
The protease core (**left**) between the domains DI and DII and hydrophobic pocket (**right**) in the domain DIV of the new CAPN1-based pramacophore models generated from the modeled bovine CAPN1/CAST4 system, superimposed on the key residues of interaction interfaces. The following pharmacophore features on the key residues (Gln109, Gly113, Lys171, and Trp298 within the protease core and Lys578, His579, Arg627, and Trp616 in the hydrophobic core of DIV) are color coded, adding location constraints to the pharmacophore features, defining the relative positions of the features required for the CAST4 inhibitor to map: H-bond donor (pointed green ball), Hydrophobic (pointed cyan ball) excluded volume (grey ball), negative charge (pointed blue ball), shape constraints (grey shape) of the interaction interface.

We selected the key residues (Gln109, Gly113, Lys171, and Trp298) in the first region considering their binding affinity correlated with the interface size and their local organization in interaction with subdomain B of CAST4. The pharmacophores for bovine CAPN1 were generated from the complex model structure with the inhibitor CAST4 as we previously predicted. The pharmacophores were constructed using the receptor-ligand pharmacophore generation protocol in DS Version 3.1 with minimum features (6) and maximum features (10). The importance of key residues in those positions can be clearly seen in Figure 13, which shows seven pharmacophore features mapped at compact regions of residues and the cavity shape of the interaction interface.

The Trp298 mutation to an alanine of bovine CAPN1 shows a highly complex destabilization (3.66 kcal/mol) due to the generation of a large cavity after alanine mutation, where there is no provision of aromatic π-π stacking interaction with two proline residues (Pro687 and Pro688 of the CAST4) and of a hydrogen bonding donor mapped to the indole nitrogen of Trp298 that corresponds to Thr685 of CAST4. The unique function of Trp298 clearly shows its importance in inhibitor interaction in two pharmacophore features, hydrogen acceptor within a distance 3.0 Å of Thr685, add excluded volume 4.0 to 5.0 Å as a large hydrophobic surface from the corresponding residue Ile686 on CAST4. The Gln109 being a large residue with neighbor Trp298 offer a side-chain allowing packing defects, is coupled with a small hydrophobic Pro687, with likely backbone H-bonding ability across the interface. The H-bonding ability of Gln109 has a smaller effect on binding affinity (substitution effect by alanine 1.59 kcal/mol), which would remain the same when mutated to alanine. Therefore, it may be less useful as a targeting key residue, but, it can be accompanied by another key residue, Trp298, providing a synergic effect for specificity of a small inhibitor. Interestingly, the Gly113 could afford conformational flexibility into the backbone and is not commonly selected as a key residue; however, it has a strong impact on CAST4 binding. The effect of mutation to alanine should be higher than 5.0 kcal/mol, and it does significantly contribute to the complex destabilization. The excluded volumes of the three key residues (Trp298, Gln109 and Gly113) at specific positions suggest that the steric barriers for small inhibitor conformers to anchor to binding sites are very important. There is a competitive binding site between the inhibitor and substrates of CAPN1 with respect to the catalytic Cys115 residue located near the center of the interface. As mentioned above, the local kink conformer of CAST4 (residues 679–686) within the site is the optimal fitting architecture characterized by complementarity both in shape and in the staggered positions against Cys115 (Figure 1 and Figure 13).

In particular, the key Gly113 residue located at the bottom of the complement pocket to the kink conformer is critical for tight packing with the inhibitor to increase binding stability by escaping the Cys115 in a hydrophilic environment. These excluded volumes, which act as another shape constraint for the inhibitor-binding, can adapt to different structural contexts in the same residues between the substance and inhibitor of CAPN1 and can be used to determine likely interaction sites for other binding partners. Moreover, the Lys171 is its basic character and is positive charged; hence, it often has a high degree of complementarity with the target if the negative charged interaction within 5.6 Å may be important for endowing with specificity for the inhibitor, alanine mutant of which reduces the complex stability by increasing mutation energy of 3.86 kcal/mol compared with the wild-type. We reconfirm the importance of key interacting residues binding to CAST4 with three mutations in the complex system (Gln109, Gly113, and Trp298 and Lys171, Gly113, and Trp298 to alanine mutations). As expected the three alanine mutations result in drastic losses (three fold, 15.01 and 14.61 kcal/mol, respectively) in binding affinity with the single mutation of Gln109Ala. In three multiple mutations, the free energy change caused by the simultaneous mutations at the residue positions in the CAPN1 is compared with the sum of the free energy change associated with single mutations at each of the residues positions. The deviations can be applied as evaluation criteria of cooperatives to improve binding affinity (The deviations of the former and the latter combinations are 4.38 and 1.71 kcal/mol, respectively, and deviations of the former combination of key residues can be more effective as targeting them with virtual small inhibitor screening). Therefore this is desirable to develop a rule-based screening function for specificity based on the features and the 3D locations.

**Figure 14:**
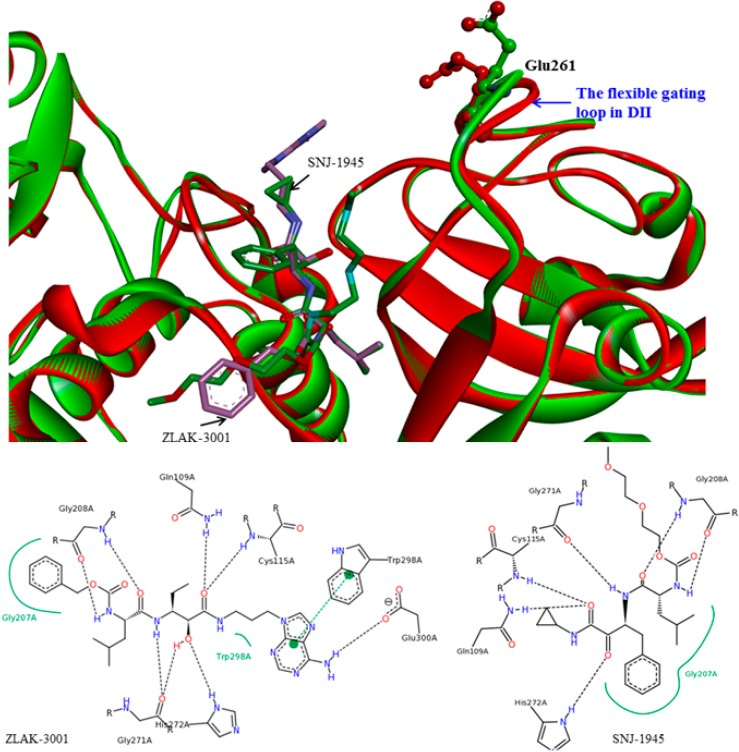
Side view of the superimposed interactions interface for the rat CAPN1proteolytic core inactivated by both ZLAK-3001 and SNJ-a945, α-ketoamide-based inhibitors-bound X-ray crystal structures in the PDB code 2R9C (red) and 2G8J (green), automatically created two-dimensional diagrams of complex with PoseView [46].

More interestingly, for these four residues, the Trp298 and Gln109 residues of CAPN1 are reported to be key residues involved in the rat CAPN1 enzyme inhibition of both α-ketoamide-based inhibitor-bound X-ray crystal structures (PDB codes 2R9C and 2G8J). Qian *et al.* [26] showed that, in two crystal structures (PDB codes 2R9C and 2R9F), the aromatic staking interaction and the H-bond between the adenine moiety of α-ketoamide inhibitor (ZLAK-3001 in PDB code 2R9C) and Trp298 and its neighboring residue Gly300 of the rat CAPN1, respectively, provided the inhibitor with a decisive potency advantage over an equivalent inhibitor (ZLAK-3002) lacking a terminal aromatic group. This revealed the importance of the aromatic stacking interactions between them. Trp298 plays a central role of transient opening of binding pockets, as found in simulations with other cysteine proteases; hence, the Trp298 residue alone is insufficient to specifically target CAPN1. SNJ-1945, another α-ketoamide-type inhibitor (in PDB code 2G8J) shares a similar interface but more localized electrostatic field binding in deeper cleft, and remarkably, it targets the same key residues (Gln109, glycine residues 207, 208, and 271, His 272, *etc.*, via H-bonding) that leupeptin uses to bind the CAPN1 protease core (as shown in Figure 6a). The cyclopropyl ring of SNJ-1945 stretches into Glu261, which is displaced in the conformation of a much more open gating loop (residues 251–271) in DII than that previously observed in 2G8J (as shown in Figure 14) from a shallow cleft formed by the indole ring of Trp298, the α carbon of Gly113, and the side chain of Gln109.

Cuerrier *et al.* [28] suggest that, the highly conserved Glu261 present across human CAPN isoforms (from CAPN1 to CAPN13 has a critical function in the CAPN group owing to its location adjacent to a non-conserved region of DII. However, in our bovine CAPN1, which is not 23-modeled structures in the isolated proteolytic core, it cannot be determined whether the contact of Glu261 contributes significantly to tight binding along with its complex with CAST4 (the Glu261Ala mutant than toward wild-type is negatively affected by −0.56 kcal/mol such as very little increased binding affinity with the CAST4). Figure 14 shows the salient features of the key residues (the Gln109 and Trp298 residues of the CAPN1) on the characterized interfaces by the rat CAPN1 and α-ketoamide-type inhibitor. The geometry of the key interacting pharmacophore features was in good agreement with the positions of the corresponding interacting residues; thus, the role of their side-chain functional groups at specific positions can be inferred from the bovine CAPN1/CAST4 complex model.

#### 2.3.2. The Definition of Allosteric Binding Site (Consisting of Lys578, His579, Trp616, and Arg627) Constraints for Virtual Small Inhibitor Screening

**Figure 15:**
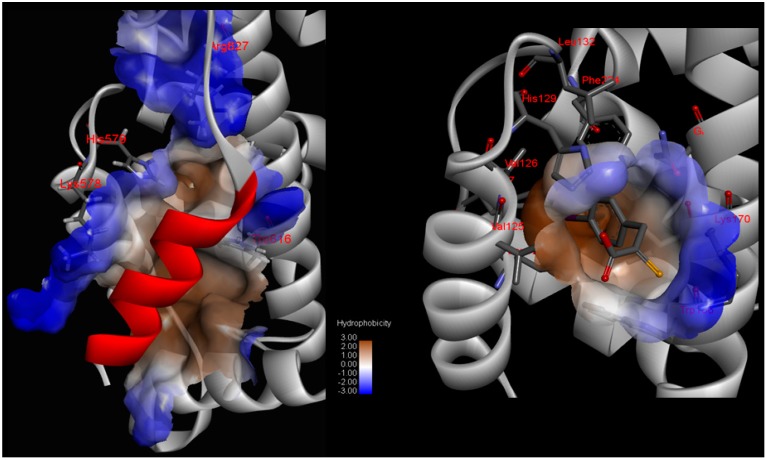
Stereo views of the hydrophobic surface plots within CAPN1 with the hydrophobic inhibitors; subdomain A of CAST4 (the natural CAPN inhibitor, **Left**) and PD150606 (the small inhibitor, **Right**) binding sites. The inhibitors bound to the hydrophobic pocket formed by EF-hands 1 and 2, that exposed on water molecules when both non-Ca^2+^-binding and Ca^2+^-binding. The inhibitors are positioned in almost the same place assuming a similar magnitude of Ca^2+^-induced conformational changes before binding to CAPN1.

It is unclear to what extent each binding contact with CAST subdomains A and B contributes to the overall free energy of binding by the relationship of structure-function alone for the 27-modeled bovine CAPN1/CAST4 system; even a binding site mutation in either region can affect the protease activity. Nevertheless, the most effective inhibition by CAST requires that all three subdomains (A, B, and C) in each inhibitory domain (CAST1 to CAST4) must bind to CAPN simultaneously for maximum inhibitory effects. To permit the coincidental binding of all subdomains to CAPN, they may also be required for other structurally well-characterized interfaces (in the hydrophobic pocket of DIV) and alternative functional epitopes (the bovine residues 639–652 of LDDALDQLSDSLGQ motif of subdomain A). Large effects (1.5 to 3.62 kcal/mol) were also seen in the hydrophobic pocket into which the conserved motif of subdomain A can put its hydrophobic residues from one surface with its hydrophilic residues fitting into exposed water molecules on the opposite face (Figure 15). The key binding residues are Lys578, His579, Trp616, and Arg627 on the hydrophobic pocket surrounded that pack together to form a tightly packed hydrophobic core from the inside. There is a hydrophobic cavity between the van der Waals surfaces of many of the polar residues that had greater mutation energies for binding affinity than the buried contact residues (Leu residues 552, 556, 623, and 631, Ile residues 571, 575, 619, and Phe612 in Figure 12). For the CAPN1-subdomain A of CAST4 interactions, these are predominantly interactions between hydrophobic interface regions; therefore, unlike the hydrophilic residues outside of the hydrophobic pocket, the patches of high surface hydrophobicity located at the central region of the interface are partially compensated by the alanine mutation, and their mutation energies were less than 0.5 kcal/mol. The most hydrophilic residues (Lys578, His579, Trp616, and Arg627, of which Trp616 contributes mainly to the binding of the CAST4 inhibitor and is most likely oriented toward the inhibitor, which is thereby accessible) on the periphery of the hydrophobic interfaces can also contribute considerably to the specificity of binding by unfavorable electrostatic interactions or steric repulsions. They may be also required for other binding factors, such as intermolecular H-bonding networks or salt-bridges. For example, Arg627 located over the center interface is capable of multiple H-bonds (up to five) and a salt-bridge out of its positive charge with contacting residues to increase binding stability by enhancing favorable interactions in the hydrophobic environment (Figure 13 and Figure 15).

The binding affinity of subdomain A to the Arg627Ala mutated enzyme was shown to decrease more than 3.5 kcal/mol compared to its wild-type. A comparison of subdomain A of CAST4 (natural enzyme inhibitor) predicted to be an amphipathic helix and PD150606 (a small inhibitor in PDB code 1NX3) showed that the two inhibitors interact with the domain DIV of CAPN1 using the same pocket residues (several bulky ring residues of Phe553, Phe612 and Trp616 and a verity of other hydrophobic residues of Leu552, Leu556, and Ile571) except for one residue, Ile576 (corresponding to Vla125 of pig CAPN1), in which they are positioned slightly differently. The hydrophobic region that settles these inhibitor molecules seems to be flexible enough that their various sizes can be accommodated as shown in Figure 15. Surprisingly, a comparison of the subset of key residues that are functionally critical for binding showed that they had the different positions according to their arrangement (Lys578, His579, Trp616 and Arg627 within bovine CAPN1 and Arg128, His129, Trp166, Lys177 within pig CAPN1) indicating different substructure of the hydrophobic region. If we use the narrowness of the hydrophobic binding site and convert the narrow binding site to shape, we will add the molecular shape constraints to the pharmacophore surrounded by the key residues (Lys578, His579, Trp616 and Arg627 of CAPN1) to increase the selectivity and ensure that reasonable sized fragments are identified as the inhibitor. The excluded volumes unlike the protease core, however, do not fully reflect the shape of the hydrophobic binding pocket in DIV that shape constants, which make contacts between the CAST4 inhibitor and the CAPN1 and are added to the pharmacophores instead of excluded volumes, in which the sizes are proportional to the number of atoms within the inhibitors (in the right of Figure 13). This should be considered since the inhibitors might impinge sterically on the CAPN1, and then the large hydrophobic region would facilitate further expansion of this binding pocket to adapt to different sized inhibitors. These shape constants also allows a greater degree of flexibility in the corresponding positioning of other pharmacophore features (H-bond acceptor, hydrophobic, and negative charged point *etc.*) from the CAPN1 key residues where those features of subdomain A of CAST4 interact. It would be reasonable to expect similar binding and induced effects from the common key residues such as His579 and Trp616 as seen in the PD150606 and pig CAPN1 complex structure (His129 and Trp166 in Figure 6b and Figure 15).

## 3. Experimental Section

### Residue Mutations and Their Impact on Protein Stability, Binding Affinity and Aggregation in the Bovine CAPN1/CAST4 Complex

In the study, we used the Protein Design protocol to predict model structures for a mutated CAPN1 (from the wild-type structure) and to calculate the energy effect of mutation for CAPN1 stability and binding affinity of CAST4 domain or Ca^2+^-ions with the corresponding single-point mutations of bovine CAPN1 itself, or of the bovine CAPN1/CAST4 complex. We were able to perform computational, amino-acid-scanning mutagenesis by mutating each residue to alanine on a set of specified CAST4 residues, which were thought to play an important role in inhibitory functions coupled with Ca^2+^-dependent anchoring to CAPN though experimental site-directed mutagenesis [37,41]. One or more mutated protein structures were created based on the wild-type structure. The side-chain conformation of the mutated residues in the protein structure was optimized by its probability and first torsion angle using a rotamer library; the probability distribution of its rotamer positions was based on experimental observation of high-resolution crystal structures [47].

The energy stabilization effect of each mutation was evaluated as the difference between the folding free energy of the mutated and wild-type structures:

$$\text{∆∆}G_{mut}=\text{∆∆}G_{folding}^{\left(mutant\right)}-\text{∆∆}G_{folding}^{\left(wild-type\right)}$$

The folding free energy (∆∆*G_folding_*) is defined as the free energy difference between the folded and unfolded states of the proteins:

∆∆*G_folding_* = ∆*G_folded_* − ∆*G_unfolded_*

The folding free energy was calculated by applying the CHARMm polar hydrogen force field [48] to the modeled protein structures. Since the bovine CAPN1 active form contains modified protonation amino acids as thiol deprotonation of Cys115 and His272 protonation at a natural pH of 7.4, the CHARMm polar hydrogen force field was applied in both the nucleophilic active site cysteine (Cys115) and its neighboring residues (including His272 and Asn296 residues). The mutation energy function also contained entropy terms of the protein side chain and backbone, which were evaluated according to room temperature in aqueous solvent. In this case, the solvent environment was implicitly considered to have a dielectric constant of 80 using the Generalized Born solvation model [49]. The total energy was calculated as an empirical weighted sum of van der Waals (E_vdw_) interaction; pH-dependent electrostatic interaction (∆*G_elec_(pH,I)*); an entropy contribution (−*TS_sc_*) related to the changes as the side-chain mobility; and a non-polar, surface dependent, contribution to solvation energy (∆*G_np_*). For this reason, the calculations of non-polar contributions to the folding free energy are related to protein ionization of the wild-type and mutated structures in both the folded and unfolded states. The electrostatic energy terms were also obtained by integration over the proton binding isotherms derived from the fractional protonation of the sites, including the effect of ionic strength (I) [50], on the calculated free energy terms:

∆*G_tot_*(*pH*) = *aE_vdw_* + ∆*G_elec_*(*pH*,*I*) − *cTS_sc_* + ∆*G_np_*

where empirical scaling parameters, a = 0.5 and c = 0.8, were applied to the terms.

The energy effect of each mutation on the binding affinity was also calculated as the difference between the binding free energy in mutated and wild-type structures, where the binding affinity of molecular partners in the bovine CAPN1/CAST4 system, or the bovine CAPN1-Ca2+ ions binding on the enzyme activation was calculated as the difference of the binding free energy (∆∆*G_bind_*) of the bound state and unbound states, $\;\text{∆∆}G_{mut}=\text{∆∆}G_{bind}^{\left(mutant\right)}-\text{∆∆}G_{bind}^{\left(wild-type\right)}$ . All interaction energy terms were calculated in the same manner as those of the pH-dependent mutation energy for bovine CAPN1 stability in the water solvent environment, while the optimal values of the other scaling factors remained the same.

Structural knowledge regarding bovine CAPN1/CAST4 interaction would identify the structural elements important for the inhibition of bovine CAPN1; therefore, the results of this study may be directly useful in screening for SNP markers of bovine CAPN1 during disease diagnosis. Activated mammalian CAPNs structures, especially the CAPN/CAST complex, have also become invaluable target models when the structure-based virtual screening of drug candidates (from discovery phase to development) is applied for over-activated CAPN, which has been linked to a variety of diseases, such as post-ischemic injury and cataract formation [33,45]. It was proposed that the effects of Ca^2+^-binding to the enzyme include activation as well as the dissociation, aggregation, and autolysis of small regular subunits [39]. Unfortunately, protease tends to aggregate when treated with divalent ions at the high-concentration required for the Ca^2+^-activated enzyme, and this could disturb the regulation of its inhibitor CAST. The inhibitor is recognized as the only structure of the activated CAPN enzyme, and it leads to a decrease in its proteolytic activity. This property of the CAPN system makes it very difficult to crystallize, not only the whole enzyme but also the enzyme/inhibitor complex. Extensive research of protein folding and protein-protein binding has shown that hydrophobic interactions play a key role in protein or antibody aggregation [39]. Indeed, the aggregation of the large CAPN catalytic subunit (four domains DI to DIV in the presence of Ca^2+^ ions shown in Figure 11) would be expected to promote exposure of hydrophobic dimerization surfaces to aqueous solvent (in particular, hydrophobic contact interfaces as fifth EF-hand motif between DIV and DVI on both large and small subunits). The dimerization interfaces in DIV and DVI have exposed hydrophobic surfaces in the same region, even in the absence of Ca^2+^ (inactivated form). This is so energetically unfavorable that the unstable effects lead to the formation of randomly associated aggregates as a result of Ca^2+^-induced conformational rearrangement and partial dissociation. Surprisingly, the crystal structures (PDB code 3BOW, 3DF0) of the rat CAPN2/CAST complex seem to be protected from the aggregation of CAPN2 resulting in encompassing of exposed hydrophobic regions on the enzyme, by the CAST inhibitor [3]. This may be a consequence of simultaneous binding to CAPN molecules by both subdomains A, and C, each of which is part of the CAST-independent-inhibitor domains (from CAST1 to CAST4) and each of which is stabilized by hydrophobic interactions in the presence of Ca^2+^ ions. As a result, there was no enzyme self-aggregation or subunit dissociation. These results provide more support for the complex model structure of bovine CAPN1/CAST4 in which self-aggregation cannot occur as a complement to CAST4 subdomain A due to through direct hydrophobic interactions with the interaction site of CAPN1. We predicted selective mutation effects and the relative importance of hydrophobic interactions on the exposed hydrophobic surfaces of the residues across the bovine CAPN1/CAST4 complex by calculating the spatial aggregation propensity (SAP) [11,40,51] based on the pre-calculated solvent accessible area (SAA) of the fully exposed side chain by the CHARMm polar hydrogen force field. The SAP for a CAPN1 mutated from the original structure was obtained as the specified radii from the hydrophobicity scale of Black and Mould [40], which was added as atom and residue properties on the patches of exposed hydrophobic residues. The hydrophobicity scale was normalized such that glycine had a hydrophobic value of zero; thus, amino acids more hydrophobic than glycine were positive, while hydrophilic residues were negative. Therefore, the bovine CAPN1 aggregation propensity for the enzyme atom is defined as:

$$\sum^{\text{​}}\left[\left(\frac{\text{SAA of side chain atoms within radius R }}{\text{SAA of side chain atoms of fully exposed residues}}\right)\times \text{residue hydrophobicity}\right]$$

The SAP for each residue on the patches of the exposed hydrophobic residues was obtained as the average of its atomic aggregation scores. High aggregation scores (0.0 < SAP < 0.5) indicated highly exposed hydrophobic regions, and then an SAP map for the region was generated by red color-coding, which allowed us to perform target mutations of those regions to prevent enzyme aggregation and thus enhance stabilization. Low SAP values (−0.5 < SAP < 0.0) indicated that the exposed protein surface was a hydrophilic region (blue). This can be expected as most of the protein surface exposed to water is usually hydrophilic. SAP might provide useful information on the physicochemical properties of interaction surfaces in the bovine CAPN1/CAST4 complex by identifying its aggregation regions. Homology modeling and all computational studies were performed with the Discovery studio (DS) 3.1 molecular modeling package [52] on a personal workstation.

## 4. Conclusions

In the absence of whole-structural data for mammalian CAPN1, we have predicted Ca^2+^-induced conformational changes before and after bovine CAPN1 activation and further the CAPN1/CAST4 complex structure by homology modeling. These structure models were refined by focusing on the binding site regions and each key interaction residues and its interaction constraints with CAST4. The focus was directed toward the relevance of trace residues (class and species-specific and conserved residues across mammalian CAPN group) and the major specificities of bovine CAPN1 with molecular properties. Our findings suggest that the positions of trace residues of bovine CAPN1 were primarily distributed from domain DII to DIII through the exposed binding grooves to water molecules and interaction regions between domains of the enzyme molecule, in which the protease activity and the binding affinity with subdomain B of CAST4 are localized to the highly flexible gating loop in DII comprising residues 251–271. In addition, they affect both the enzyme itself and the complex with CAST inhibitor stabilities rather than functionality (as binding affinity with its substrate or inhibitor or Ca^2+^ ions). The hydrophobic interfaces with high SAP are the main factors for enzyme stability and aggregation. In this observation, the effects of trace residues of bovine CAPN1 were also confirmed by the fact that CAPN1/CAST4 complex stability has a greater impact on the enzyme origin of disease mutations (R385H, D600G related with the LGMD-2A) than the binding affinity with CAST4. Furthermore, for all of trace residues analyzed our combined site-directed alanine mutagenesis studies and pharmacophore models revealed the importance of key residues within each binding site region for CAPN1-CAST4 interactions. These results provide more support for the interpretation of the requirements for CAPN1-small inhibitor interactions based on their X-ray crystal complex structures. One of the most intriguing findings of our studies is whether the geometry of the key interacting pharmacophore features or the shape constraints present in the binding site cavities were all previously unknown to create the specificities of the CAPN1 subgroup. Most importantly, this is based on the finding that key residues of CAPN1 [53] show no apparent sequence preferences compared to CAPN2 in agreement with their pharmacophore features, but the depth and breadth of the CAPN1 active site were well reflected in the geometry between their pharmacophore features, which correlates with CAPN inhibitor specificities that are conformation dependent. In this paper, we have presented a novel approach whereby inhibition can arbitrate some of the key residues within bovine CAPN1 directly interacting with its endogenous inhibitor, CAST, the alanine mutations of which in these contact regions significantly reduce not only the binding affinity for the inhibitory domains of CAST but also their complex stability, less effective than the complete protease-protease inhibitor interactions did. These key residues can be targeted in the virtual screening of competitive small inhibitors, which mimic the natural inhibitor CAST throughout fingerprint fragments with its important properties, with the large polypeptide substrates against the CAPN1 subgroup with improved specificity and selectivity as compared to the wild-type inhibitor. The bovine CAPN1/CAST complex models, which are subjects to analysis in the study, have been deposited in the Protein Model Database [54] and anyone can accessible to the public (PMDB ID: PM0079218, PM0079221, and PM007922 in both inactive and active model structures of bovine CAPN1 and in the Ca^2+^-bounded complex of two subdomains CAST4, respectively).
